# Statistical analysis plan for the randomized controlled trial Tenecteplase in Wake-up Ischaemic Stroke Trial (TWIST)

**DOI:** 10.1186/s13063-022-06301-0

**Published:** 2022-05-19

**Authors:** Agnethe Eltoft, Tom Wilsgaard, Melinda B. Roaldsen, Mary-Helen Søyland, Erik Lundström, Jesper Petersson, Bent Indredavik, Jukka Putaala, Hanne Christensen, Janika Kõrv, Dalius Jatužis, Stefan T. Engelter, Gian Marco De Marchis, David J. Werring, Thompson Robinson, Arnstein Tveiten, Ellisiv B. Mathiesen

**Affiliations:** 1 Department of Neurology, University Hospital of North Norway, Tromsø, Norway; 2 Department of Clinical Medicine, UiT The Arctic University of Norway, Tromsø, Norway; 3 Department of Community Medicine, UiT The Arctic University of Norway, Tromsø, Norway; 4 Department of Clinical Research, University Hospital of North Norway, Tromsø, Norway; 5 Department of Neurology, Sorlandet Hospital Kristiansand, Kristiansand, Norway; 6 Department of Neuroscience, Neurology, Uppsala University, Uppsala, Sweden; 7 Department of Neurology, Skåne University Hospital, Malmö, Sweden; 8 Department of Medicine, St. Olavs Hospital, Trondheim University Hospital, Trondheim, Norway; 9 Department of Neuromedicine and Movement Science, Norwegian University of Science and Technology, Trondheim, Norway; 10 Department of Neurology, Helsinki University Hospital and University of Helsinki, Helsinki, Finland; 11 Department of Neurology, Bispebjerg Hospital and University of Copenhagen, Copenhagen, Denmark; 12 Department of Neurology and Neurosurgery, University of Tartu and Tartu University Hospital, Tartu, Estonia; 13 Department of Neurology and Neurosurgery, Center for Neurology, Vilnius University, Vilnius, Lithuania; 14 Department of Neurology, University Hospital of Basel and University of Basel, Basel, Switzerland; 15 Neurology and Neurorehabilitation, University Department of Geriatric Medicine Felix Platter, University of Basel, Basel, Switzerland; 16 Stroke Research Centre, UCL Queen Square Institute of Neurology, London, UK; 17 College of Life Sciences and NIHR Biomedical Research Centre, University of Leicester, Leicester, UK

**Keywords:** Ischemic stroke, Thrombolysis, Wake-up stroke, Tenecteplase, TWIST, Acute stroke therapy

## Abstract

**Background:**

Patients with wake-up ischemic stroke are frequently excluded from thrombolytic treatment due to unknown symptom onset time and limited availability of advanced imaging modalities. The Tenecteplase in Wake-up Ischaemic Stroke Trial (TWIST) is a randomized controlled trial of intravenous tenecteplase 0.25 mg/kg and standard care versus standard care alone (no thrombolysis) in patients who wake up with acute ischemic stroke and can be treated within 4.5 h of wakening based on non-contrast CT findings.

**Objective:**

To publish the detailed statistical analysis plan for TWIST prior to unblinding.

**Methods:**

The TWIST statistical analysis plan is consistent with the Consolidating Standard of Reporting Trials (CONSORT) statement and provides clear and open reporting.

**Discussion:**

Publication of the statistical analysis plan serves to reduce potential trial reporting bias and clearly outlines the pre-specified analyses.

**Trial registration:**

ClinicalTrials.govNCT03181360. EudraCT Number 2014-000096-80. WHO ICRTP registry number ISRCTN10601890.

**Supplementary Information:**

The online version contains supplementary material available at 10.1186/s13063-022-06301-0.

## Introduction

Patients with wake-up ischemic stroke are traditionally ineligible for thrombolytic treatment due to an unknown time of symptom onset. Recent randomized controlled trials have shown that thrombolytic treatment may be safe and effective in wake-up stroke patients selected by findings on advanced imaging such as MRI and CT perfusion [1–3]. However, these imaging modalities are not readily available 24/7 at all hospitals. The Tenecteplase in Wake-up Ischaemic Stroke Trial (TWIST) is an international, investigator-initiated, multi-center, prospective, randomized controlled, open-label, blinded end-point, superiority trial of intravenously administered tenecteplase 0.25 mg/kg and standard care versus standard care alone (i.e. no thrombolysis) in patients who wake up with acute ischemic stroke and can be treated within 4.5 h of wakening. Patients are selected based on the findings of non-contrast computer tomography (NCCT) and randomized (1:1) to intravenous (IV) thrombolysis with tenecteplase or no thrombolysis. Both treatment arms will receive best standard care, including endovascular interventions for proximal cerebral artery occlusion, where indicated. The study is recruiting patients from 10 countries. Based on a revised sample size estimation in June 2020, the aim was to include 600 patients (300 in each treatment arm).

A description of the TWIST trial protocol has been published previously [4]. The purpose of this statistical analysis plan (SAP) is to outline in detail the pre-determined plan for the statistical analyses associated with the primary report of the trial results prior to unblinding and analysis of the trial primary and key secondary outcomes. The TWIST SAP is consistent with the Consolidating Standard of Reporting Trials (CONSORT) statement [5]. The SAP has been finalized prior to completion of the data collection and will be adhered to in analyzing data. The plan for all subsequent secondary analyses and content of all subsequent publications cannot be specified in detail at present, but where appropriate, we set out the general analytical approach.

### Aims and hypothesis

The primary aim of TWIST is to test the hypothesis that treatment with IV tenecteplase within 4.5 h of wakening is superior to standard care (no IV thrombolysis) and improves functional outcome at 3 months in patients with wake-up ischemic stroke selected by NCCT brain imaging.

### Patient population

Patients aged 18 years and older with new stroke symptoms acquired during sleep and a clinical diagnosis of stroke with limb weakness and National Institute of Health Stroke Scale (NIHSS) score ≥3, or aphasia, are eligible for inclusion. Detailed inclusion and exclusion criteria have been published previously [4] and are listed in the supplementary material. In brief, the main exclusion criteria are NIHSS score >25 or NIHSS consciousness score >2, infarct size larger than >1/3 of the middle cerebral artery territory, or intracranial hemorrhage on NCCT.

### Randomization

A remote, web-based, computer-generated randomization procedure is used. All online submissions are secured by the use of password site entry and data encryption procedures via the TWIST online webpage. Investigators record patient details via a secure web interface before randomization takes place. The randomization procedure includes a standard minimization algorithm which ensures that the treatment groups are balanced across all centers in all countries for key prognostic factors: age, NIHSS score, and time since wake-up. Patients are allocated with a probability of 0.80 to the treatment group which minimizes the difference between the groups with regards to the key prognostic factors. Patients will be randomly allocated to open-label IV tenecteplase plus best standard care or to best standard care alone (i.e., no IV thrombolysis).

### Study procedures

Details on the background and study design are documented in the published trial protocol [4]. Both patients and investigators are unblinded to treatment allocation. Primary outcome assessments are performed by telephone interview at 3 months (90±7 days) by trained study personnel, blinded to treatment allocation. The results of 3-month follow-up interviews are securely stored and kept separated from the investigators.

Only the Data Monitoring Committee (DMC) has access to interim data and results. An independent statistician, who otherwise is not involved in the trial, prepares biannual unblinded reports of the interim data and results to the DMC. The DMC will work on the principle that a difference of at least 3 standard errors in an interim analysis of a major outcome event may be needed to justify halting, or modifying the study before the end of planned recruitment (Haybittle-Peto rule) [6]. This criterion has the practical advantage that the exact number of interim analyses would be of little importance, and so no fixed schedule is proposed. A less-strict rule might be used if there was evidence that tenecteplase was unsafe. If the DMC judges credible evidence of harm, or overwhelming evidence of efficacy, the committee will advise the chairman of the Steering Committee. Unless this happens, the Steering Committee will remain blinded to the interim results.

### Trial objectives and outcomes

The primary outcome is functional outcome at 3 months on an ordinal scale (0–6), defined by the modified Rankin Scale (mRS) score. The use of a categorical shift in mRS at 3 months after stroke has been advocated to provide a more comprehensive index of the clinical impact of acute stroke treatment [7]. This accounts for the fact that thrombolytic treatment is often not completely curative, but has the potential to improve patient outcome over the whole range of functional measurements. Table 1 outlines all relevant outcomes for the primary report.

**Table 1:** Objectives and outcomes of the Tenecteplase in wake-up ischaemic stroke trial

Objectives	Outcome
**Primary**	
Improve functional outcome in participants with wake-up ischemic stroke	Functional outcome defined as shift across the ordinal modified Rankin Scale (mRS) (0–6) at 3 months follow-up
**Secondary**	
Increase the proportion of patients with excellent functional outcome	Proportion of participants free from disability defined as functional outcome mRS score of 0–1 at 3 months follow-up
Increase the proportion of patients with good functional outcome	Proportion of participants functionally independent defined as an mRS score 0–2 at 3 months follow-up
Increase the proportion of patients with response to treatment stratified by baseline stroke severity	Proportion of patients with response to treatment; mRS 0 for patients with a mild deficit at study entry (NIHSS <=7), mRS 0-1 for patients with a moderate deficit (NIHSS 8-14), and mRS 0–2 for patients with a severe deficit (NIHSS >14)
Reduce mortality rate	Proportion of participant mortality over the 3 months study period
Determine safety based on the rate of symptomatic intracranial hemorrhage (SICH)	Proportion of patients with SICH as defined by the SITS-MOST criteria [8] Proportion of patients with SICH as defined by the IST-3 criteria [9]
Determine safety based on the rate of parenchymal hemorrhage type 2 (PH-2) [10]^a^	Proportion of patients with parenchymal hemorrhage type 2 on follow-up imaging at 24 (± 6) h
Determine safety based on the rate of any intracranial hemorrhage	Proportion of patients with any intracranial hemorrhage detected on follow-up imaging at 24 (± 6) h
Reduce poor functional outcome or death	Proportion of patients with mRS score of 4–6 at 3 months

^a^Hematoma occupying 30% or more of the infarcted tissue, with obvious mass effect [10]

Additional clinical, radiological, and health care system-related outcomes are included in the supplemental material. Details about definition and adjudication of clinical events and expert imaging readings are also listed in the supplementary material and Additional file 1.

### Sample size considerations

The primary endpoint in TWIST is mRS across the full ordinal scale (shift analysis). We originally based our sample size estimation on the results of a Cochrane systematic review of the effect of rt-PA within 4.5 h of stroke onset, assessed as a binary endpoint (favorable outcome mRS 0–2 versus mRS 3-6) [11]. As a sample size estimation based on ordinal logistic regression analysis is more appropriate, we revised the sample size estimation in 2020. The revised sample size estimation was based on observations from recent studies of thrombolytic treatment in patients with wake-up stroke. In the largest randomized controlled trial of wake-up stroke, WAKE-UP^1^, the difference between thrombolysed and non-thrombolysed patients was 11.5% for a favorable outcome defined as mRS 0-1. A difference of 11.5% was also found in a recent meta-analysis [12] of six observational studies on patients with unknown stroke onset time, where the favorable outcome was defined as mRS 0–2. The MRI-based inclusion criteria in WAKE-UP compared to the CT-based inclusion in TWIST could lead to a smaller treatment effect in TWIST. We assume a treatment effect of 10% absolute difference in a binary endpoint setting (mRS 0–1 versus mRS 2–6) and a distribution between mRS categories similar to that of the WAKE-UP trial [1]. Accordingly, we anticipated a favorable outcome in 42% of participants in the non-thrombolysed group versus 52% in the thrombolysed group, which corresponds to an odds ratio (OR) of 1.50, and mRS distribution across the control group in six levels (categories 5 and 6 merged) of 15%, 27%, 23%, 17%, 13%, and 5%, respectively. With a power of 80%, a two-sided significance level of 5%, and an effect size specified as an OR of 1.50 from an ordinal logistic regression model for the ordinal outcome in the control group, the estimated sample size was 600. The revised target was therefore increased from 500 to 600 patients, i.e., 300 patients in each treatment arm.

## Statistical analysis plan

### Analysis principles and general considerations

All analyses will be conducted on data from all randomly assigned patients according to the intention to treat (ITT) principle, i.e. patients will be analyzed in the group they were randomized to, no matter what treatment they received, and regardless of whether they deviated from the protocol in any way. All outcomes and analyses are prospectively categorized as primary, secondary, or exploratory.

Ordinal logistic regression is pre-specified as the method for the analysis of the primary outcome using the common odds ratio as the corresponding effect size measure, restricting adjustment to age, symptom severity (baseline NIHSS score), and time from wake-up to randomization. All covariate adjustment variables are continuous variables, assumed linearity and non-linearity allowed. Non-linearity will only be included if the addition of the squared variable significantly improves the model fit (judged by an improvement in the likelihood ratio test).

Differences in all outcomes between the two treatment groups will be tested independently at the two-tailed 0.05 level of significance. All estimates of treatment effects will be presented with 95% confidence intervals (CIs). No formal adjustments will be undertaken to reduce the overall type I error associated with both secondary and exploratory analyses including the subgroup analyses. Their purpose is to supplement evidence from the primary analysis to better characterize the treatment effect. Results from the secondary and exploratory analyses will be interpreted in this context. Pre-specified subgroup analyses (as outlined in the supplementary material) will be carried out irrespective of whether there is a significant treatment effect on the primary outcome.

Sensitivity analyses for the primary outcome will be undertaken to test the robustness of the primary analysis with regards to protocol violations, baseline imbalance, clustering effects, and missing data (details in supplementary material). Unadjusted analysis will be undertaken as sensitivity analysis and will be presented for all primary and secondary outcomes.

Analyses will be conducted primarily using SAS 9.4 statistical software. Proposed tables and figures for the main publication are presented in Additional file 2.

### Treatment of missing values

Rigorous efforts are made to minimize the amount of missing outcome data. Minimal loss to follow-up for the 3 months assessment of the primary outcome is anticipated. If functional status at 3 months is unknown for any patient, we will apply the following algorithm: If the patient was alive at 3 months and measurements are available after baseline, we will use the level of function recorded on day 7 (i.e. measured at day 7 or prior to discharge from hospital) to impute functional status at 3 months. Hence, 3 months mRS will be imputed for patients with status at day 7 or discharge. We have chosen this simple form of single imputation, as it usually classifies patients appropriately, where both day 7 and 3 months data are known, and any additional gain from more complex multiple imputation methods is likely to be small [13]. The mRS outcome is assumed to be missing-at-random. Important explanatory and auxiliary variables such as age, baseline NIHSS score, geographical region, time of randomization after wake-up and treatment group are being collected, and will be examined to assess the plausibility of the missing at random assumption. No missing data are expected in adjustment covariates as completeness is assured by the web based randomization procedure. Any missing components of the NIHSS score will be imputed by multiple imputation. Sensitivity analyses based on different hypothesis about the missingness pattern of the primary outcome will be conducted to test for the robustness of the primary outcome, including analysis of the “complete case population,” i.e., based on the data of completers, using only observed without accommodating missing data.

### Trial profile

Flow of patients through the study will be displayed in a standard Consolidated Standards of Reporting Trials diagram. The report will include the number of patients included, withdrawn, lost to follow-up, the number who received the allocated treatment and the number of patients analyzed.

### Patient characteristics and baseline comparisons

To assess balance, description of collected baseline characteristics will be presented for the tenecteplase and control groups. Discrete variables will be summarized as frequencies and percentages. Unless otherwise indicated, percentages will be calculated according to the number of patients for whom data are available. If there are more than 5% missing values for a variable, the denominator will be added as a footnote in the corresponding summary table. Continuous variables will be summarized using either mean and standard deviation, or median and interquartile range (IQR). Time intervals will be summarized by medians and IQRs. Some of these variables were used in the minimization algorithm determining randomization allocation, and good balance would thus demonstrate successful operation of the algorithm.

#### Primary outcome: Differences in 3 months functional outcome across the full mRS scale between treatment groups

##### Outcome

This outcome measure will occur at 3 months follow-up. The outcome is measured using the mRS ordinal scale (range 0–6).

##### Main analysis

Common OR from an ordinal logistic regression model adjusted for age, stroke severity (baseline NIHSS score) and time since wake-up will be used if the proportional odds assumptions are satisfied (approximate likelihood-ratio test of proportionality of odds are not significant). However, if the proportional odds assumptions are not satisfied, the assumption-free Wilcoxon–Mann–Whitney Generalized Odds Ratios (WMW GenOR) will be used [14].

##### Statistical hypotheses

The null hypothesis which is to be refuted in the ordinal logistic regression is that the common OR is equal to 1, i.e., there is no difference in treatment effect between the intervention and control groups. The null hypothesis to be refuted in WMW GenOR test is equality of ranks when ties are split evenly. The null hypothesis for the WMW GenOR test states that the probability that the treatment observation is better than the control observation is the same as the probability that the treatment observation is worse than the control observation (splitting the ties equally), i.e., the WMW GenOR is equal to 1.

##### Analysis of the primary outcome

If the analyses of the baseline characteristics of the trial patients show clear differences in key prognostic factors (age, stroke severity, and time since wake-up) between treatment groups, this may complicate the estimation of the treatment effect. The primary analysis of the effect of treatment on the primary outcome will therefore be adjusted for the following covariates: age, symptom severity (baseline NIHSS score), and time since wake-up. An unadjusted analysis will also be presented. A separate set of analyses will be performed stratified for patients who received endovascular treatment and those that did not.

#### Secondary efficacy and safety outcomes: Differences in proportions of patients with clinical outcomes between treatment groups

##### Outcomes

- *Excellent functional outcome (mRS 0–1) at 3 months poststroke:*

Outcome is measured using mRS dichotomized by excellent functional outcome (mRS 0-1) versus unfavorable (mRS 2–6) outcome.

- *Good functional outcome (mRS 0–2) at 3 months poststroke:*

Outcome is measured using the mRS dichotomized by good (mRS 0-2) versus unfavorable (mRS 3–6) outcome.

- *The proportion of patients with response to treatment on day 7 (or discharge):*

Response to treatment is defined as mRS 0 for patients with mild deficit at study entry (NIHSS <=7), mRS 0–1 for patients with moderate deficit (NIHSS 8-14), and mRS 0-2 for patients with severe deficit (NIHSS >14).

- *Symptomatic intracranial hemorrhage as defined by Safe Implementation of Thrombolysis in Stroke-Monitoring Study (SITS-MOST)*^*8*^
- *Symptomatic intracranial hemorrhage as defined by defined by International Stroke Trial-3 (IST-3)*^*9*^
- *Parenchymal hemorrhage type 2*^*10*^ *on follow-up imaging at 24±6 h*
- *Any intracranial hemorrhage detected on follow-up imaging at 24±6 h*
- *Poor outcome defined as mRS score* dichotomized by poor outcome (mRS 4-6) versus mRS *0–3 at 3 months*

These outcomes are each measured on a binary scale and are defined as positive in the presence of event and negative otherwise.

##### Statistical hypotheses

For each separate outcome, the set of statistical hypotheses are p (IV tenecteplase) = *p* (control) versus *p* (IV tenecteplase) ≠ *p* (control), where *p* (IV tenecteplase) is the proportion of subjects with the specific clinical outcome in the IV tenecteplase group and *p* (control) is the proportion with the specific clinical outcome in the control group. If OR=1, the treatment effect is equal in both groups.

##### Analysis method

An unconditional logistic regression model will be fitted for each outcome separately to estimate the OR associated with treatment effect, restricting adjustment to age, baseline NIHSS score, and time since wake-up. Corresponding 95% CIs will be provided. For the outcome *proportion of patients with response to treatment on day 7*, no adjustment for baseline NIHSS will be performed. Unadjusted analyses will also be presented for all secondary efficacy and safety outcomes. A separate set of analyses will be performed stratified by patients who received endovascular treatment and those that did not.

#### Differences in 3 months mortality between treatment groups

##### Outcome

This secondary safety outcome is measured as time-to-event data. Event is defined as death of any cause within 3 months post-stroke.

##### Statistical hypothesis

The set of statistical hypotheses is hazard (IV tenecteplase) = hazard (control) versus hazard (IV tenecteplase) ≠ hazard (control). If HR=1, the hazard of death is equal in both groups.

##### Analysis method

The effect of treatment allocation on survival will be assessed using time-to-event analysis by Cox proportional hazards models adjusted for age, baseline NIHSS score and time since wake-up. The risk related to treatment will be presented as HRs with the corresponding 95% CI and corresponding survival plots will be presented. Proportional hazard assumption will be tested on the basis of Schoenfeld residuals. If the proportional hazard assumption is not met, survival time will be split in three intervals of 30 days and a hazard ratio will be estimated for each interval using a time-dependent Cox model. Unadjusted Kaplan-Meier survival curves with the log-rank test will also be presented. A separate set of analyses will be performed stratified for patients who received endovascular treatment and those that did not.

Exploratory analyses for additional outcomes, planned subgroup and meta-analyses are included in the supplementary material

#### Trial status

Recruitment started in June 2017 and ended with a total of 578 included patients on September 30th, 2021. Collection of primary endpoints were ongoing until January 2022.

#### Summary and conclusions

TWIST will provide evidence on whether wake-up ischemic stroke patients can be treated safely and effectively with tenecteplase 0.25 mg/kg within 4.5 h of awakening based on the findings of non-contrast CT.

## Supplementary Information

**Additional file 1.** List of imaging variables in the tenecteplase in wake up ischeamic stroke trial (TWIST).

**Additional file 2.** Proposed tables and figures for main publication of the tenecteplase in wake up ischeamic stroke trial (TWIST).

**Additional file 3.**

## Data Availability

Once completed, TWIST will contribute data to a planned future individual patient data meta-analysis. Data may be made available upon request.

## Abbreviations

TWIST: Tenecteplase in Wake-up Ischaemic Stroke Trial
NCCT: Non-contrast computer tomography
SAP: Statistical analysis plan
IV: Intravenous
NIHSS: National Institute of Health Stroke Scale
DMC: Data Monitoring Committee
mRS: Modified Rankin Scale
SICH: Symptomatic intracranial hemorrhage
PH-2: Parenchymal hemorrhage type 2
ITT: Intention to treat
OR: Odds ratio
CI: Confidence intervals
IQR: Interquartile range
WMW GenOR: Wilcoxon–Mann–Whitney Generalized Odds Ratios
HR: Hazard ratio
