# Supplementary material for: Statistical analysis plan for the randomized controlled trial Tenecteplase in Wake-up Ischaemic Stroke Trial (TWIST)
Source: Trials. 2022 May 19;23:421. doi: 10.1186/s13063-022-06301-0 (PMC9118782; doi:10.1186/s13063-022-06301-0)
Supplement: Supplementary file 1 — Additional file 1. List of imaging variables in the tenecteplase in wake up ischeamic stroke trial (TWIST). [file 13063_2022_6301_MOESM1_ESM.docx]

## List of imaging variables in the tenecteplase in wake up ischeamic stroke trial (TWIST)

### **Non-contrast CT Day 1 and Day 2:**

**Ischemic changes/signs (Y=yes/N=no)**

- Presence (Y/N)
  - If “yes” classify
    - Site (cortical, periventricular/lacunar, borderzone, brainstem/cerebellar)
    - Side (L/R)
    - Does hypoattenuation involve > 1/3 of the MCA territory (Y/N)

**ASPECT Score**- anterior circulation

|  | **NCCT** |
| --- | --- |
| **Region** | **Hypoattenuation** |
| [caudate](https://radiopaedia.org/articles/caudate-nucleus?lang=us) |  |
| [putamen](https://radiopaedia.org/articles/putamen?lang=us) |  |
| [internal capsule](https://radiopaedia.org/articles/internal-capsule?lang=us) |  |
| [insular cortex](https://radiopaedia.org/articles/insular-cortex?lang=us) |  |
| M1: "anterior MCA cortex," corresponding to frontal operculum |  |
| M2: "MCA cortex lateral to insular ribbon" corresponding to anterior temporal lobe |  |
| M3: "posterior MCA cortex" corresponding to posterior temporal lobe |  |
| M4: "anterior MCA territory immediately superior to M1" |  |
| M5: "lateral MCA territory immediately superior to M2" |  |
| M6: "posterior MCA territory immediately superior to M3" |  |
| **ASPECT total** | **10 minus number of affected regions­­ = ­­___** |

- **Hyperdense artery**
  - Presence (Y/N)
    - If “yes” classify
    - side (L/R)
      - segments (M1 prox/dist, M2, A1,ICA supra-/infraclinoid, vertebro-basilar, other
- **ICH**
  - Presence (Y/N)
  - If “yes” state
    - side (L/R)
    - site (cortical, periventricular/lacunar, borderzone, brainstem/cerebellar)
    - volume
    - Hemorrage type (HI1/HI2/PH1/PH2/PH remote/IVH/SAH/SDH)

### **Non-contrast CT Day 1 only**

- **Prior stroke lesions**
  - presence of well-circumscribed hypodense area without any mass effect or edema (Y/N)
  - if “yes” state
    - side (L/R)
    - site (cortical, periventricular/lacunar, borderzone, brainstem/cerebellar)
    - probable old hemorrhage (Y/N)
- **Leukoaraiosis (baseline)**
  - Presence (Y/N)
  - van Swieten score (0-4)
- **Atrophy/reduction in brain tissue volume**
  - Presence (Y/N)
  - If “yes” classify as
    - central (none/mild/moderate)
    - cortical (none/mild/moderate)
- **Non-stroke lesions**
  - Presence (Y/N)
  - If “yes” state
    - side (L/R)
    - site
    - lesion type (cerebral tumour, cerebral abcess, cyst, other)

### **Non-contrast CT Day 2 only**

Volume of ischemic lesion (mL)

### **CTA Day 1 and Day 2**

- **Artery occlusion**
  - Presence (Y/N)
  - If “yes” state
    - side (L/R)
    - location (MCA; M1 prox/dist, M2, ACA; A1, ICA supra-/infraclinoid, extracranial carotid artery, PCA, vertebro-basilar)
    - length of clot (measured within the arterial tree in the axial CT angiography source images)
- **Collateral status** (CT angiography Collateral Score)
  - 0: absent collaterals in >50% of the occluded territory
  - 1: diminished collaterals in >50% of the occluded territory
  - 2: diminished collaterals in <50% of the occluded territory
  - 3: collaterals equal to or more than the contralateral hemisphere.
- **Degree of obstruction/recanalisation** (revised TICI)
  - 0: no perfusion or anterograde flow beyond site of occlusion
  - 1: recanalisation without reperfusion. Contrast penetration exists past the initial obstruction but with minimal filling of the normal territory
  - 2: recanalisation with incomplete reperfusion [<50% (grade 2a), ≥50% (grade 2b), near-complete (grade 2c)]
  - 3: recanalisation with complete reperfusion
- **ASPECT Score**- anterior circulation

|  | **CTA source images** |
| --- | --- |
| **Region** | **Hypoattenuation** |
| [caudate](https://radiopaedia.org/articles/caudate-nucleus?lang=us) |  |
| [putamen](https://radiopaedia.org/articles/putamen?lang=us) |  |
| [internal capsule](https://radiopaedia.org/articles/internal-capsule?lang=us) |  |
| [insular cortex](https://radiopaedia.org/articles/insular-cortex?lang=us) |  |
| M1: "anterior MCA cortex," corresponding to frontal operculum |  |
| M2: "MCA cortex lateral to insular ribbon" corresponding to anterior temporal lobe |  |
| M3: "posterior MCA cortex" corresponding to posterior temporal lobe |  |
| M4: "anterior MCA territory immediately superior to M1" |  |
| M5: "lateral MCA territory immediately superior to M2" |  |
| M6: "posterior MCA territory immediately superior to M3" |  |
| **CTASI total** | **10 minus number of affected regions­­ = ­­___** |

- **pc- ASPECT – posterior circulation (CT angiography source imaging)**

|  | **CTA source images** |
| --- | --- |
| **Region** | **Hypoattenuation** |
| Left or right thalamus, cerebellum or PCA-territory, respectively (1 point); any part of midbrain or pons (2 points). |  |
| [Thalamus](https://radiopaedia.org/articles/putamen?lang=us): left, right (1 point for each) |  |
| Cerebellum or PCA-territory: (1 point for each) |  |
| Midbrain or pons - any part of (2 points). |  |
| **pc-ASPECT total** | **10 minus points at affected regions­­ = ­­___** |

**CTP day 1**

- Perfusion lesion volume by different definitions (time to maximum (T max)>2,4,6 seconds and time to peak (TTP)>2,4,6 seconds)
- Ischemic core volume by different definitions (cerebral blood flow (CBF) <30% and CBF <20%)
- Penumbral volume
  - perfusion lesion minus ischemic core according to varying definitions
- Mismatch
  - Presence (Y/N)
  - If “yes” state
    - Volume (mL)
    - Percentage

### **MRI at day 1 and day 2**

- Acute DWI lesion with corresponding low signal on ADC map
  - Presence (Y/N)
  - If “yes” state
    - side (L/R)
    - site (cortical, periventricular/lacunar, borderzone, brainstem/cerebellar)
    - volume(mL)
- FLAIR lesion
  - Presence (Y/N)
  - If “yes” state
    - side (L/R)
    - site
    - volume(mL)
- Presence of DWI/Flair mismatch (Y/N)
- MRI-ASPECT and pcASPECT scores

**Prior stroke lesions**

- - presence (Y/N)
  - if “yes” state
    - side (L/R)
    - site (cortical, periventricular/lacunar, borderzone, brainstem/cerebellar)
    - Probable old hemorrhage? (Y/N)

**Leukoaraiosis**

- - Presence (Y/N)
  - van Swieten score (0-4)

**Atrophy/reduction in brain tissue volume**

- - Presence Y/N
  - If yes classify as
    - Central (none/mild/moderate)
    - Cortical (none/mild/moderate)
- **Non-stroke lesions**
  - Presence Y/N
  - If “yes” state:
    - side (L/R)
    - site
    - Lesion type (cerebral tumor, cerebral abscess, cyst, other)
- **ICH**
  - Presence Y/N
  - If “yes” state:
    - side (L/R)
    - site
    - size
    - type (HI1/HI2/PH1/PH2/PH remote/IVH/SAH/SDH)

**Artery occlusion**

- - Presence (Y/N)
  - If “yes” state;
    - side (L/R)
    - location
      - MCA; M1 prox/dist, M2
      - ACA; A1
      - ICA supra-/infraclinoid, extracranial carotid artery
      - PCA
      - vertebro-basilar
    - length of clot (measured within the arterial tree in the axial CT angiography source images)
    - Collateral status

**Degree of obstruction/recanalisation** (revised TICI)

- - 0: no perfusion or anterograde flow beyond site of occlusion
  - 1: recanalization without reperfusion. Contrast penetration exists past the initial obstruction but with minimal filling of the normal territory
  - 2: recanalization with incomplete reperfusion [<50% (grade 2a), ≥50% (grade 2b), near-complete (grade 2c)]
  - 3: recanalization with complete reperfusion
