# Supplementary material for: Statistical analysis plan for the randomized controlled trial Tenecteplase in Wake-up Ischaemic Stroke Trial (TWIST)
Source: Trials. 2022 May 19;23:421. doi: 10.1186/s13063-022-06301-0 (PMC9118782; doi:10.1186/s13063-022-06301-0)
Supplement: Supplementary file 2 — Additional file 2. Proposed tables and figures for main publication of the tenecteplase in wake up ischeamic stroke trial (TWIST). [file 13063_2022_6301_MOESM2_ESM.docx]

## Proposed tables and figures for main publication of the tenecteplase in wake up ischeamic stroke trial (TWIST)

##
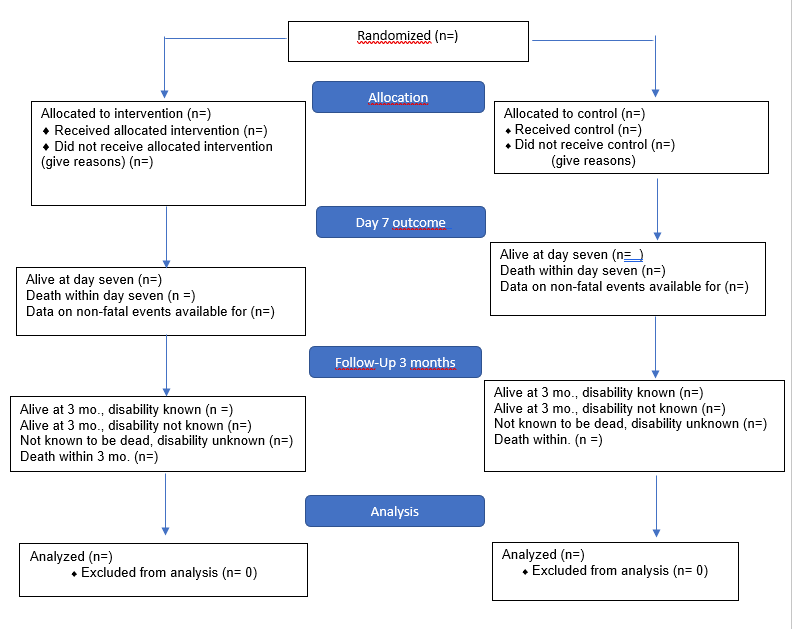


**Figure 1. Consort 2010 Flow diagram**

| **Table 1. Characteristics of Patients at Baseline^a^** | | | | | | |
| --- | --- | --- | --- | --- | --- | --- |
|  |  | |  | **Tenecteplase (n=?)** |  | **Control (n=?)** |
|  | Age-yr | |  |  |  |  |
|  |  | Mean (SD) |  |  |  |  |
|  |  | Median (IQR) |  |  |  |  |
|  | Age groups (years) | |  |  |  |  |
|  |  | <60 |  |  |  |  |
|  |  | 60-79 |  |  |  |  |
|  |  | ≥80 |  |  |  |  |
|  | Sex | |  |  |  |  |
|  |  | Women |  |  |  |  |
|  |  | Men |  |  |  |  |
|  | Country— no. (%) | |  |  |  |  |
|  |  | Norway |  |  |  |  |
|  |  | Sweden |  |  |  |  |
|  |  | Denmark |  |  |  |  |
|  |  | Finland |  |  |  |  |
|  |  | Estonia |  |  |  |  |
|  |  | Latvia |  |  |  |  |
|  |  | Lithuania |  |  |  |  |
|  |  | United Kingdom |  |  |  |  |
|  |  | Switzerland |  |  |  |  |
|  |  | New Zealand |  |  |  |  |
|  | Final diagnosis at discharge — no. (%) | |  |  |  |  |
|  |  | Definite ischemic stroke |  |  |  |  |
|  |  | Probable ischemic stroke |  |  |  |  |
|  |  | Other diagnosis |  |  |  |  |
|  | Stoke risk factors and medical history— no. (%) | |  |  |  |  |
|  |  | Hypertension |  |  |  |  |
|  |  | Diabetes mellitus |  |  |  |  |
|  |  | Atrial fibrillation |  |  |  |  |
|  |  | Active smoker |  |  |  |  |
|  |  | Previous stroke or TIA |  |  |  |  |
|  |  | Coronary artery disease |  |  |  |  |
|  |  | Current use of an anticoagulant agent |  |  |  |  |
|  |  | Current use of an antiplatelet agent |  |  |  |  |
|  | Pre-morbid modified Rankin Scale score | |  |  |  |  |
|  |  | 0 |  |  |  |  |
|  |  | 1 |  |  |  |  |
|  |  | 2 |  |  |  |  |
|  | Median NIHSS score (IQR)^b^ | |  |  |  |  |
|  |  | Mild (0- 7) |  |  |  |  |
|  |  | Moderate (8–14) |  |  |  |  |
|  |  | Severe (≥15) |  |  |  |  |
|  | Endovascular treatment— no. (%) | |  |  |  |  |
|  | Median time from last known to be well to randomisation — (IQR)- min | |  |  |  |  |
|  | Median time from wake-up to randomisation — (IQR) - min | |  |  |  |  |
|  | Median time from wake-up to hospital admission (IQR) - min | |  |  |  |  |
|  | Median time from hospital arrival to initiation of therapy (IQR) - min | |  |  |  |  |

^a^Values are means ±standard deviations(SD). IQR denotes interquartile range.

^b^Scores on the National Institutes of Health Stroke Scale (NIHSS) range from 0 (normal) to 42 (death), with higher scores indicating greater deficit.


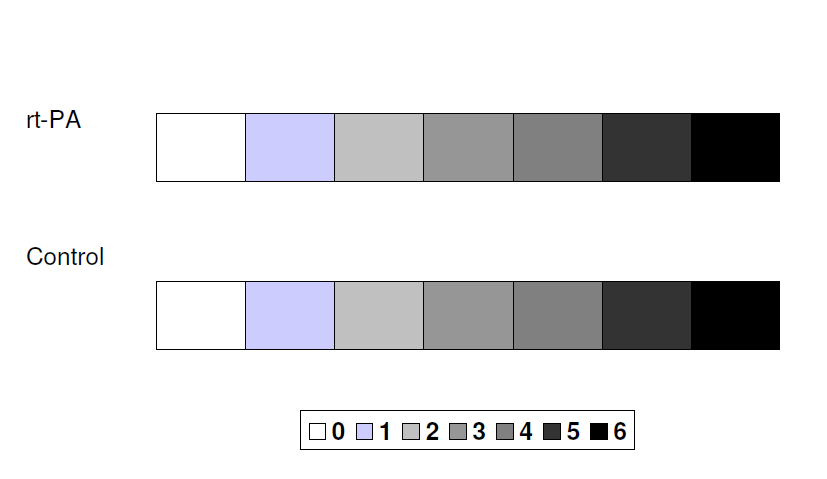


**Figure II Bar chart showing the distribution of mRS scores in each treatment group**

| **Table 2. Efficacy and safety outcomes (intention to treat population)^a^** | | | | | | | | | | | | | | |
| --- | --- | --- | --- | --- | --- | --- | --- | --- | --- | --- | --- | --- | --- | --- |
| **Outcome** | | |  | **Tenecteplase**  **(n=?)** |  | **Control**  **(n=?)** |  | **Unadjusted Effect Size^h^ (95% CI)** |  | **P Value** |  | **Adjusted Effect Size^h^ (95% CI)** |  | **P Value** |
| **Primary efficacy outcome** | | |  |  |  |  |  |  |  |  |  |  |  |  |
|  | Score on the modified Rankin scale at 3 months^b^ | |  |  |  |  |  |  |  |  |  |  |  |  |
|  |  | 0 |  |  |  |  |  |  |  |  |  |  |  |  |
|  |  | 1 |  |  |  |  |  |  |  |  |  |  |  |  |
|  |  | 2 |  |  |  |  |  |  |  |  |  |  |  |  |
|  |  | 3 |  |  |  |  |  |  |  |  |  |  |  |  |
|  |  | 4 |  |  |  |  |  |  |  |  |  |  |  |  |
|  |  | 5 |  |  |  |  |  |  |  |  |  |  |  |  |
|  |  | 6 |  |  |  |  |  |  |  |  |  |  |  |  |
|  | Functional improvement^c^ | |  |  |  |  |  |  |  |  |  |  |  |  |
| **Secondary efficacy outcomes** | | |  |  |  |  |  |  |  |  |  |  |  |  |
|  | Excellent functional outcome at 3 months^d^ | |  |  |  |  |  |  |  |  |  |  |  |  |
|  | Good functional outcome^e^ | |  |  |  |  |  |  |  |  |  |  |  |  |
|  | Response to treatment^f^ | |  |  |  |  |  |  |  |  |  |  |  |  |
| **Safety outcomes** | | |  |  |  |  |  |  |  |  |  |  |  |  |
|  | Death within 3 months after intervention | |  |  |  |  |  |  |  |  |  |  |  |  |
|  | Symptomatic intracranial hemorrhage | |  |  |  |  |  |  |  |  |  |  |  |  |
|  |  | As defined by SITS- MOST |  |  |  |  |  |  |  |  |  |  |  |  |
|  |  | As defined by IST-3 |  |  |  |  |  |  |  |  |  |  |  |  |
|  | Parenchymal hemorrhage type 2 | |  |  |  |  |  |  |  |  |  |  |  |  |
|  | Any intracranial haemorrhage | |  |  |  |  |  |  |  |  |  |  |  |  |
|  | Poor functional outcome or death^g^ | |  |  |  |  |  |  |  |  |  |  |  |  |

^a^Adjusted analyses included age, baseline NIHSS score and time since wake-up as covariates.

^b^Scores on the modified Rankin scale range from 0 to 6, with 0 indicating no neurologic deficit, 1 no clinically significant disability (return to all usual activities), 2 slight disability (able to handle own affairs without assistance but unable to carry out all previous activities), 3 moderate disability requiring some help (e.g., with shopping, cleaning, and finances but able to walk unassisted), 4 moderately severe disability (unable to attend to bodily needs without assistance and unable to walk unassisted), 5 severe disability (requiring constant nursing care and attention), and 6 death.

^c^Functional improvement was defined as an improvement of at least 1 point on the modified Rankin scale at 3 months and was assessed as a common odds ratio in an ordinal logistic-regression analysis.

^d^Excellent functional outcome was defined as a score of 0 to 1 on the modified Rankin scale at 3 months.

^e^Good functional outcome as a score of 0 to 2 on the modified Rankin scale at 3 months.

^f^Response to treatment is defined as mRS 0 for patients with mild deficits at study entry (NIHSS <=7), mRS 0-1 for patients with moderate deficits (NIHSS 8-14), and mRS 0-2 for patients with severe deficits (NIHSS >14).

^g^Poor functional outcome defined as patients with mRS score of 4-6 at 3 months.

^h^Effect sizes are assessed as odds ratios, except for death within 3 months assessed as hazard ratios. The 95% confidence intervals for the secondary outcomes were not adjusted for multiple comparisons.

| **Table 3. Efficacy and safety outcomes (intention to treat population) stratified according to thrombectomy treatment^a^** | | | | | | | | | | | | |
| --- | --- | --- | --- | --- | --- | --- | --- | --- | --- | --- | --- | --- |
|  | | |  | **Patients not treated with thrombectomy** | | | |  | **Patients treated with thrombectomy** | | | |
| **Outcome** | | |  | **Tenecteplase**  **(n=?)** | **Control**  **(n=?)** | **Adjusted Effect Size^h^ (95% CI)** | **P-Value** |  | **Tenecteplase**  **(n=?)** | **Control**  **(n=?)** | **Adjusted Effect Size^h^ (95% CI)** | **P -Value** |
| **Primary efficacy outcome** | | |  |  |  |  |  |  |  |  |  |  |
|  | Score on the modified Rankin scale at 3 months^b^ | |  |  |  |  |  |  |  |  |  |  |
|  |  | 0 |  |  |  |  |  |  |  |  |  |  |
|  |  | 1 |  |  |  |  |  |  |  |  |  |  |
|  |  | 2 |  |  |  |  |  |  |  |  |  |  |
|  |  | 3 |  |  |  |  |  |  |  |  |  |  |
|  |  | 4 |  |  |  |  |  |  |  |  |  |  |
|  |  | 5 |  |  |  |  |  |  |  |  |  |  |
|  |  | 6 |  |  |  |  |  |  |  |  |  |  |
|  | Functional improvement^c^ | |  |  |  |  |  |  |  |  |  |  |
| **Secondary efficacy outcomes** | | |  |  |  |  |  |  |  |  |  |  |
|  | Excellent functional outcome at 3 months^d^ | |  |  |  |  |  |  |  |  |  |  |
|  | Good functional outcome^e^ | |  |  |  |  |  |  |  |  |  |  |
|  | Response to treatment^f^ | |  |  |  |  |  |  |  |  |  |  |
| **Safety outcomes** | | |  |  |  |  |  |  |  |  |  |  |
|  | Death within 3 months after intervention | |  |  |  |  |  |  |  |  |  |  |
|  | Symptomatic intracranial hemorrhage | |  |  |  |  |  |  |  |  |  |  |
|  |  | As defined in SITS- MOST |  |  |  |  |  |  |  |  |  |  |
|  |  | As defined in IST-3 |  |  |  |  |  |  |  |  |  |  |
|  | Parenchymal hemorrhage type 2 | |  |  |  |  |  |  |  |  |  |  |
|  | Any intracranial haemorrhage | |  |  |  |  |  |  |  |  |  |  |
|  | Poor functional outcome or death^g^ | |  |  |  |  |  |  |  |  |  |  |

^a^Adjusted analyses included age, baseline NIHSS score and time since wake-up as covariates.

^b^Scores on the modified Rankin scale range from 0 to 6, with 0 indicating no neurologic deficit, 1 no clinically significant disability (return to all usual activities), 2 slight disability (able to handle own affairs without assistance but unable to carry out all previous activities), 3 moderate disability requiring some help (e.g., with shopping, cleaning, and finances but able to walk unassisted), 4 moderately severe disability (unable to attend to bodily needs without assistance and unable to walk unassisted), 5 severe disability (requiring constant nursing care and attention), and 6 death.

^c^Functional improvement was defined as an improvement of at least 1 point on the modified Rankin scale at 3 months and was assessed as a common odds ratio in an ordinal logistic-regression analysis.

^d^Excellent functional outcome was defined as a score of 0 to 1 on the modified Rankin scale at 3 months.

^e^Good functional outcome as a score of 0 to 2 on the modified Rankin scale at 3 months.

^f^Response to treatment is defined as mRS 0 for patients with mild deficits at study entry (NIHSS <=7), mRS 0-1 for patients with moderate deficits (NIHSS 8-14), and mRS 0-2 for patients with severe deficits (NIHSS >14).

^g^Poor functional outcome defined as patients with mRS score of 4-6 at 3 months.

^h^Effect sizes are assessed as odds ratios, except for death within 3 months assessed as hazard ratios. The 95% confidence intervals for the secondary outcomes were not adjusted for multiple comparisons.

**Figure III**

Kaplan Meier survival plot tenecteplase treated patients versus controls.
