# Supplementary material for: Statistical analysis plan for the randomized controlled trial Tenecteplase in Wake-up Ischaemic Stroke Trial (TWIST)
Source: Trials. 2022 May 19;23:421. doi: 10.1186/s13063-022-06301-0 (PMC9118782; doi:10.1186/s13063-022-06301-0)
Supplement: Supplementary file 3 — Additional file 3. [file 13063_2022_6301_MOESM3_ESM.docx]

# Supplemental Material for Statistical analysis plan for the Tenecteplase in Wake-up Ischaemic Stroke Trial (TWIST)

## Trial Registration EudraCT Number, 2014-000096-80. ClinicalTrials.gov, NCT03181360. Registered June 8, 2017.

## Inclusion and exclusion criteria

Complete list of inclusion criteria

• Stroke symptoms on awakening that were not present before sleep

• Clinical diagnosis of stroke with limb weakness and National Institutes of Health Stroke Scale (*NIHSS)* score ≥3, or aphasia

• Treatment with tenecteplase is possible within 4.5 hours of awakening

• Written consent from the patient, non-written consent from the patient (witnessed by non-participating health care personnel), or written consent from the nearest family member

Complete list of exclusion criteria

• Age <18 years

- NIHSS score >25 or NIHSS consciousness score >2, or seizures during stroke onset
- Findings on non-contrast CT (NCCT) that indicate that the patient is unlikely to benefit from treatment:
  - Infarction comprising more than >1/3 of the middle cerebral artery territory on NCCT or CT perfusion
  - Intracranial hemorrhage, structural brain lesions which can mimic stroke (e.g. cerebral tumor)
- Active internal bleeding or high risk of bleeding, e.g.:
  - Major surgery, trauma or gastrointestinal or urinary tract hemorrhage within the previous 21 days, or arterial puncture at a non-compressible site within the previous 7 days
  - Any known defect in coagulation, e.g., current use of vitamin K antagonist with an INR >1.7 or prothrombin time >15 seconds, or use of direct thrombin inhibitors or direct factor Xa inhibitors during the last 24 hours (unless reversal of effect can be achieved by agents such as idarusizumab) or with elevated sensitive laboratory tests (such as aPTT, INR, platelet count, eucarin clotting time, TT, or appropriate factor Xa activity assays), or heparins during the last 24 hours or with an elevated aPTT greater than the upper limit of normal
  - Known defect of clotting or platelet function or platelet count below 100,000/mm^3^ (patients on antiplatelet agents can be included)
  - Ischemic stroke or myocardial infarction in previous 3 months, previous intracranial hemorrhage, severe traumatic brain injury or intracranial or intraspinal operation in previous 3 months, or known intracranial neoplasm, arteriovenous malformation or aneurysm
- Contraindications to tenecteplase, e.g., acute bacterial endocarditis or pericarditis; acute pancreatitis; severe hepatic dysfunction, including hepatic failure, cirrhosis, portal hypertension; active hepatitis; systemic cancer with increased bleeding risk; hemostatic defect including secondary to severe hepatic, renal disease; organ biopsy; prolonged cardiopulmonary resuscitation > 2 min (within 2 weeks)
- Persistent blood pressure elevation (systolic ≥185 mmHg or diastolic ≥110 mmHg), despite blood pressure lowering treatment
- Blood glucose 20.0 mmol/L (use of finger-stick measurement devices is acceptable)
- Pregnancy, positive pregnancy test, childbirth during last 10 days, or breastfeeding. In any woman of childbearing potential, a pregnancy test must be performed and the result assessed before trial entry
- Other serious or life-threatening disease before the stroke: severe mental or physical disability (e.g. Mini Mental Status score<20, or mRS score ≥3, or life expectancy less than 12 months
- Patient unavailability for follow-up (e.g. no fixed address)

## Adjudication and definitions of clinical outcomes

All safety outcomes will be adjudicated by an independent endpoint adjudication committee. For each patient who is reported to have deteriorated clinically or to have developed symptoms suggesting that a new cerebral event has occurred within 7 days of randomization, the adjudication committee will review selected data from the day 7 form and other appropriate source data and the expert panels blinded reading of any brain images taken between randomization and discharge. The adjudication committee will be provided data on the date and time of randomization, the patients neurological symptoms (NIHSS score) at entry, the date and time of any event and the suspected type of event, and a detailed report of the expert’s opinion of any post-randomization images. These data will be reviewed blinded to the treatment allocation.

**Death:** If the patient has died by day 7, death will be classified according to cause:

1. Initial stroke
2. Recurrent stroke
3. Myocardial infarction
4. Infection
5. Other vascular cause (e.g. systemic emboli)
6. Other non-vascular cause (e.g. malignancy, trauma)
7. Unknown cause

**Symptomatic intracranial hemorrhage (SICH):**

The IST-3 and SITS-MOST definitions of symptomatic intracranial hemorrhage are used:

***SICH per IST-3***^4^: symptomatic intracranial hemorrhage is defined as clinically significant deterioration (neurological deterioration, new headache, new acute hypertension, new nausea or vomiting, or sudden decrease in conscious level) or death within the first 7 days of treatment with evidence of either significant brain parenchymal hemorrhage (local or distant from the infarct) or significant hemorrhagic transformation (HT) of an infarct on brain imaging. This definition is inclusive, maximizing the sensitivity to any risk related to bleeding associated with thrombolytic treatment.^5^
***SICH per SITS-MOST***^6^: SICH is defined as local or remote parenchymal hematoma type 2 on the imaging scan obtained 22-36 hours after treatment, plus neurological deterioration, as indicated by an increase in NIHSS score of ≥4 points compared to baseline value or the lowest value between baseline and 24 (± 6) hours, or hemorrhage leading to death*.*^6^ This definition is conservative and ensures the inclusion of only clinically relevant large intracranial hemorrhages likely to be responsible for a worsening of functional outcome.^5^

### **Recurrent stroke:**

Neurological deterioration (increase of ≥2 on NIHSS, after exclusion of other causes for neurological deterioration) occurring after 72 hours will be considered as a recurrent stroke. Recurrent stroke will be classified as ischemic, hemorrhagic or unknown (if not documented on imaging).

**Neurological deterioration due to index stroke**:

The definition is an increase of ≥ 2 points in one or more of the NIHSS sub-scores (irrespective of improvement in any of the other NIHSS). When diagnosing neurological deterioration due to index stroke, systemic reasons for deterioration, such as drug-induced hypotension, drug-induced drowsiness, and intercurrent disease should be excluded. No significant hemorrhage should be found on post randomization CT or MR scan.

**Acute myocardial infarction:**

Either one of the following criteria satisfies the diagnosis of myocardial infarction:

1. Typical rise and gradual fall (troponin) or more rapid rise and fall (CK-MB) of biochemical markers of myocardial necrosis with at least one of the following:
   1. ischemic coronary symptoms;
   2. development of pathologic Q waves on the ECG;
   3. ECG changes indicative of ischemia (ST segment elevation or depression); or
   4. coronary artery intervention (e. g. coronary angioplasty)
2. Autopsy pathologic findings of an acute myocardial infarction.

**Major extracranial bleeding:**

Definition of major extracranial bleeding: Clinically overt bleeding associated with one or more of:

- Transfusion of >2 red cell units of blood
- A decrease in hemoglobin of 20 g/l (=2 g/dl, = 1.24 mmol/l)
- Bleeding into retroperitoneum, intraocular space or major joint
- Bleeding leading to permanent treatment cessation

## Additional outcomes

*Clinical outcomes:*

- NIHSS score at 24 (± 6) hours and day 7 (or discharge whichever came first)
- Change in NIHSS score from baseline to 24 (± 6) hours and from baseline to day 7 (or discharge)
- Proportion of patients with NIHSS score reduction > 8 points or reaching 0 to 1 at 24 (± 6) hours and at day 7 (or discharge, whichever comes first)
- Mini-Mental State Exam MMSE (telephone version) score at 3 months
- EuroQol score (EQ-5D-3L), both the summary index and the proportion of patients with good quality of life according to prespecified cut-off (EQ-5D Index ≥70)
- Proportion of patients with
  - Stroke progression
  - Recurrent ischemic stroke
  - Major extracranial bleeding
  - Myocardial infarction

*Radiological outcomes:*

- Infarct volume measured on CT or MRI at 24 (± 6) hours
- Alberta Stroke Program Early Computed Tomography (ASPECT) score^7^ at 24 (± 6) hours
- Posterior circulation Acute Stroke Prognosis Early CT Score (pc-ASPECTS) score^8^ at 24 (± 6) hours
- Change in ASPECT score and pc- ASPECT score at 24 (± 6) hours (absolute and relative to baseline) ^9^
- Recanalization at 24 (± 6) hours according to mTICI 2b/3^10^ in patients with proximal arterial occlusion at baseline
- Reperfusion at 24 (± 6) hours of baseline ischemic penumbra in patients with penumbra imaging at baseline

*Use of health care system resources:*

- Length of hospital stay
- Nursing home care after discharge
- Re-hospitalization during first 3 months

## Planned exploratory analyses

NIHSS score at 24 (± 6) hours and day 7 (Absolute and change from baseline to follow-up and NIHSS score reduction > 8 points or reaching 0 to 1). As appropriate, analysis of covariance and unconditional logistic regression models will be fitted to estimate the beta coefficient and OR, respectively, associated with treatment effect on NIHSS score. Death will be awarded the worst score of 42. Treatment group is an independent variable and NIHSS score reduction of >8 points or reaching 0 to 1 at 24 (± 6) hours or day 7 (dichotomized) or change in NIHSS score (range from 0 (normal) to 42 (most severe)) is the dependent variable, including age, time since wake up and side of infarct as a covariate for adjustment purposes. In analysis of covariance, NIHSS score at follow-up is the dependent variable and baseline NIHSS is included as a covariate for adjustment purposes.

Infarct volume on CT or MRI brain imaging 24 (± 6) hours will be examined using quantile and linear regression, with treatment group as an independent variable and infarct volume as the dependent variable. Distribution will be assessed with appropriate transformations (e.g., cubic root transformation) for linear regression analysis. ASPECT/ pc-ASPECTS score at 24 (± 6) hours will be examined using multivariable ordinal logistic regression. Image modality (CT or MRI) and baseline ASPECT/pc-ASPECTS score will be included as a covariate for adjustment purposes as appropriate.

## Recanalization on CT angiography (dichotomized as < and ≥50% reperfusion) at 24 (± 6) hours poststroke will be assessed by an unconditional logistic regression model fitted to estimate the OR associated with treatment effect, including site of arterial occlusion as a covariate for adjustment purposes. This analysis will be restricted to patients with initial vessel occlusion in CTA and scanned with repeat CTA at 24 hrs.

Treatment effect on health-related quality of life (EuroQol) (a) If the proportional odds assumptions are satisfied EuroQol (in each dimension) will be assessed by common OR from an ordinal logistic regression model adjusted for age, baseline NIHSS score and time since wake-up. (b) If the proportional odds assumptions are not satisfied, assumption free WMW GenOR will be used. For reaching the prespecified cut-off outcome (EQ-5D Index ≥70), an unconditional logistic regression model will be fitted for each outcome separately to estimate the OR associated with treatment effect.

## Imaging assessments

All patients will undergo NCCT and CT angiography (CTA) if possible before randomization, and NCCT again at 24±6 hours post randomization. CTA at 24±6 hours will only be performed in patients with a positive finding on CTA before randomization. CT perfusion (CTP) and MRI are sometimes performed prior to randomization at centers where these imaging modalities are available. In addition, a repeat NCCT scan is required if the patient deteriorates neurologically or intracranial hemorrhage is suspected for any reason. Although NCCT scanning is the preferred examination for 24±6 hours follow-up, MRI brain imaging is allowed. All scans are transferred to a cd and sent to the Trial Coordinating Centre at the University Hospital of North Norway. Images are assessed with all original identifiers stripped from the record, and then viewed via a secure web-based image viewing system by the panel of expert radiologists at the Melbourne Brain Centre under the leadership of Andrew Bivard and Mark Parsons. All assessments are made blind to all patient details and treatment allocation. Manual and semi-automated image assessments are performed as specified in Appendix 1. Analyses of the imaging data aim to evaluate the influence on risks and benefits of tenecteplase related to acute ischemic lesion extent (NCCT and ASPECT, CTASI ASPECT, PC ASPECT scores, CTP penumbra and infarct core) and background brain features (leukoaraiosis, prior lacunes and cortical infarcts) in the context of multivariable prediction models incorporating key clinical variables. Particular interest will focus on the effect of baseline findings suggestive of penumbra on CTP or presence of DWI/FLAIR mismatch on baseline MRI, to predict benefit of tenecteplase in patients in the subgroup where MRI or CTP assessments at admission are available.

## Planned subgroup analyses

Planned subgroup analyses are listed below. These subgroups were selected after review of factors that are predictors of prognosis, and for which there is prior evidence that they are potentially important effect modifiers. All groups will be studied for interaction with the effect of tenecteplase on the primary outcome and each of the prespecified secondary outcomes, controlling for imbalances in baseline characteristics. The 2-way interactions between treatment groups (tenecteplase or control) and the predefined demographic, clinical and imaging variables on the primary outcome will be explored through multivariable ordinal logistic regression for the primary outcome. For each treatment-by-subgroup interaction a likelihood ratio test will be used with appropriate degrees of freedom.

If appropriate, additional analyses of subgroups will be performed. This will generate a large number of exploratory analyses. The interpretation will depend on the p-value for interaction, and the size and confidence limits for the effects in the subgroups being compared. Forest plots will be constructed to illustrate subgroup analyses. Similar analyses will be performed for secondary outcomes with the corresponding analytical approach outlined above.

## Pre-specified subgroups

- Age (< 60, 60-79, ≥80 years)
- Sex
- Time from wake-up to randomization (0-1.5 h, 1.5-3 h, 3-4.5 h)
- Baseline stroke severity (NIHSS <8, 8-14 and ≥15)
- Geographic region
- Baseline systolic blood pressure (< 120, 120-139, 140-179, ≥180),
- Prior smoking
- Prior diabetes mellitus
- Prior antiplatelet treatment
- Prior anticoagulant treatment
- History of atrial fibrillation
- Oxfordshire Community Stroke Project classification; total anterior circulation infarct (TACI), partial anterior circulation infarct (PACI), posterior circulation infarct (POCI), or lacunar infarct (LACI) based on their maximum neurological defects)
- Imaging findings

- the appearance of the acute lesion

- - visible lesion (yes(y)/no(n))
  - ASPECTS score (≤7 versus > 7) and (≤5 versus >5)
  - Collateral Score (CT angiography Collateral Score ≤1 versus >1)
  - Hyperdense artery on NCCT/proximal artery occlusion on CTA (y/n)

- location of infarct (lacunar, cortical, infratentorial)

- background brain features (leukoaraiosis evaluated by van Swieten scale^11^ and prior vascular lesions)

## Sensitivity analysis

Sensitivity analyses for the primary outcome will be performed to test for the robustness of the primary analysis with regards to protocol violations, baseline imbalance, clustering effects and missing data as outlined below. All individuals are included in sensitivity analyses.

Adherence to the intervention will be reported. To account for effects of any off-protocol interventions, the results will also be reported for the “per protocol population” who received tenecteplase compared with the group who received standard care (no iv thrombolysis) “per protocol”. For safety outcomes sensitivity analysis will be performed and reported for the “safety population” in which a patient is included if, and only if, they actually received a study treatment.

If analyses of the baseline characteristics of the patients in the trial show clear differences in key prognostic factors (age, stroke severity and time since wake-up) between treatment groups, this may complicate the estimation of the effect of treatment. Both baseline covariate-adjusted and unadjusted results will be reported, however adjusted analysis is pre-specified as the primary analysis for this RCT.

Sensitivity analyses with regard to clustering effects will be conducted by calculating p-values and CIs for the treatment effect on the primary outcome after adjustment for (i) center (taken as a random effect); (ii) country (taken as a random effect); and (iii) region. These analyses will be performed using mixed effect ordered logistic regression models and treatment effects will be expressed as adjusted common ORs. In the case that centers with significant data quality issues are identified, further sensitivity analyses will be run to assess whether adjustment for these factors affects the primary outcome. Similar analyses will be performed for the secondary outcomes.

## Protocol Deviations

The nature and reasons for the protocol violation shall be recorded in the eCRF, in the source documents and in the monitoring visit report. All such serious non-compliance will be followed up and reported per local regulations. In parallel, corrective and/or preventive actions are undertaken and documented, including any retraining of the investigator and site staff. All patients who have been included in the trial will be followed up, irrespective of whether treatment was discontinued prematurely, or whether the protocol was violated.

Protocol deviations in consent procedure

These will be tabulated and accompanied by a brief textual description.

Protocol deviations at infusion

It is possible that some patients allocated to tenecteplase will not receive their allocated treatment or receive incorrect dose, and some of those allocated control will receive thrombolysis. These deviations will be tabulated. Patients will remain in their allocated treatment group for analysis, irrespective of treatment received.

## Data Management

Database lock and unblinding will be performed after collection of all primary outcomes, final monitoring visits and data quality control have been finalized. The data management plan will be available on the twist portal webpage <https://twist.uit.no/portal/>. The Trial Master File and Statistical Master File are securely stored de-identified with an individual code for each participant at the sponsors secure research server, password-protected and accessible only to specified study personnel.

## Systematic reviews and meta-analyses

The findings regarding the effects of treatment on the components of the primary outcome and the key secondary outcomes will be presented in the context of an update of a systematic review to give an overall meta-analytic assessment of the effects of recanalization treatment in wake-up stroke. Currently ongoing randomized trials of tenecteplase in acute ischemic stroke are targeted to include altogether more than 9,000 patients in different time windows and clinical scenarios,^13^ and TWIST will contribute data to a planned future individual patient data meta-analysis of all these trials.

## References

1. Wardlaw JM, Murray V, Berge E, et al. Recombinant tissue plasminogen activator for acute ischaemic stroke: an updated systematic review and meta-analysis. *Lancet* 2012; 379: 2364-2372. 2012/05/29. DOI: 10.1016/s0140-6736(12)60738-7.

2. Thomalla G, Simonsen CZ, Boutitie F, et al. MRI-Guided Thrombolysis for Stroke with Unknown Time of Onset. *N Engl J Med* 2018; 379: 611-622. 2018/05/17. DOI: 10.1056/NEJMoa1804355.

3. Zhu RL, Xu J, Xie CJ, et al. Efficacy and Safety of Thrombolytic Therapy for Stroke with Unknown Time of Onset: A Meta-Analysis of Observational Studies. *J Stroke Cerebrovasc Dis* 2020; 29: 104742. 2020/03/05. DOI: 10.1016/j.jstrokecerebrovasdis.2020.104742.

4. Whiteley W, Lindley R, Wardlaw J, et al. Third International Stroke Trial. *Int J Stroke* 2006; 1: 172-176. DOI: 10.1111/j.1747-4949.2006.00043.x.

5. Kummer Rv, Broderick JP, Campbell BCV, et al. The Heidelberg Bleeding Classification. *Stroke* 2015; 46: 2981-2986. DOI: doi:10.1161/STROKEAHA.115.010049.

6. Wahlgren N, Ahmed N, Davalos A, et al. Thrombolysis with alteplase for acute ischaemic stroke in the Safe Implementation of Thrombolysis in Stroke-Monitoring Study (SITS-MOST): an observational study. *Lancet* 2007; 369: 275-282. 2007/01/30. DOI: 10.1016/s0140-6736(07)60149-4.

7. Barber PA, Demchuk AM, Zhang J, et al. Validity and reliability of a quantitative computed tomography score in predicting outcome of hyperacute stroke before thrombolytic therapy. ASPECTS Study Group. Alberta Stroke Programme Early CT Score. *Lancet* 2000; 355: 1670-1674. 2000/07/25. DOI: 10.1016/s0140-6736(00)02237-6.

8. Puetz V, Sylaja PN, Coutts SB, et al. Extent of Hypoattenuation on CT Angiography Source Images Predicts Functional Outcome in Patients With Basilar Artery Occlusion. *Stroke* 2008; 39: 2485-2490. DOI: doi:10.1161/STROKEAHA.107.511162.

9. Mokin M, Primiani CT, Siddiqui AH, et al. ASPECTS (Alberta Stroke Program Early CT Score) Measurement Using Hounsfield Unit Values When Selecting Patients for Stroke Thrombectomy. *Stroke* 2017; 48: 1574-1579. 2017/05/11. DOI: 10.1161/strokeaha.117.016745.

10. Zaidat OO, Yoo AJ, Khatri P, et al. Recommendations on angiographic revascularization grading standards for acute ischemic stroke: a consensus statement. *Stroke* 2013; 44: 2650-2663. 2013/08/08. DOI: 10.1161/strokeaha.113.001972.

11. van Swieten JC, Hijdra A, Koudstaal PJ, et al. Grading white matter lesions on CT and MRI: a simple scale. J. Neurol. Neurosurg. Psychiatry 1990; 53: 1080-1083. 1990/12/01. DOI: 10.1136/jnnp.53.12.1080.

12. Senn S. Statistical Issues in Drug Development. 2nd ed. Chichester: John Wiley, 2008, pp. p. 173.

13. Putaala J, Saver JL, Nour M, et al. Should Tenecteplase be Given in Clinical Practice for Acute Ischemic Stroke Thrombolysis? *Stroke* 2021; 52: 3075-3080. DOI: doi:10.1161/STROKEAHA.121.034244.
